# Practical Medicine and Therapeutics

**Published:** 1837-04

**Authors:** 


### PRACTICAL MEDICINE AND THERAPEUTICS.

### *Remarkable Case of Spontaneous Combustion.* By Dr. Joly.

Bernard, æt. 73, and his wife, æt. 65, have long indulged to excess in spirit drinking. September 6th, they both became intoxicated, remained alone the whole night, and were found dead on the following morning. Four hours after they were found dead, Dr. Joly and the "Procureur du roi" went to see the bodies. The room which contained them was shut. Several pieces of furniture in it were covered with a grey soot. There was a strong empyreumatic smell, and on the floor, between a table covered with bottles and glasses, which had contained brandy, and the cinders of an extinguished fire, lay the legs of the two corpses and a shapeless carbonaceous mass. Two of the legs, belonging to the same individual, had on stockings of black wool and cloth slippers. One of the stockings only was burned at its upper part. The skin covered by the slippers was but reddened; the tissues beneath, when cut into, presented no peculiar appearance. An inch above the knees, the thighs were reduced to a black, shapeless, carbonaceous mass. There were no traces of the external genitals. Of the pelvis, and the parts contained within its cavity, there remained but the calcined superior edge of the left ilium, and the enlarged left ovary buried in the midst of oily and fetid carbon. There was a separation of the articulation of the lumbar vertebræ; and at this part the body was divided into two, in consequence of having rested upon the other situated beneath it. Two or three vertebræ, which thus became exposed to the air, were consequently calcined and whitened. These were quite distinct from a mass of spongy and shining carbon, corresponding to the thoracic cavity and its contents. The only portion of this which was at all solid was the vertebral column, to which was still attached some blackened fragments of the first ribs of the left side. The calcined cervical vertebræ terminated in the incinerated cranium, which was so extremely friable as almost to fall into dust on the endeavour being made to lift it. The lower jaw alone had preserved more consistence. Beneath the remains of this corpse, and forming an X with it, were those of the second. The left leg, naked and covered with vesicles containing a reddish serum on its anterior surface, was burned to the bone the whole of its length, posteriorly. It was disconnected with the body. A cat had bitten the muscles of the calf, and torn them to the extent of several inches. A fatty and disagreeable liquid oozed from this laceration. The right leg was burned like the left. Its whole anterior surface was covered with large phlyctenæ, as well as the sole of the foot, although the latter entirely rested upon the ground. About three inches above the knees, the thighs were converted into a heap of black and unctuous carbon. The pelvic region had disappeared. At the part where the former corpse lay across the latter, the clothes were strongly adherent to the remains of the bodies, in consequence of the slowness of the combustion. In the different layers of charcoal which were interposed, it was easy to recognize the character of the garments. The right lung and the liver could be recognized. They had lost about half their size; their surface was hard, varnished, and brittle; when cut, their consistence was that of soft cheese; but the texture of the liver was closer and more homogeneous than that of the lung. The vertebral column and ribs consisted of a more compact carbon than that which was formed by the soft parts. About an inch and a half from the hearth was an entire and sooty head. The prominence of the nose and the orbital cavities were still marked. This bony box was broken by the slightest shock, and in the middle of its cavity, resting on the foramen magnum, was the dried brain, about the size of a hen's egg. Of the superior extremities of these two corpses, a few inches of one calcined humerus, and three united and calcined metacarpal bones, only were found. Allowance being made for those parts which had undergone slight alteration with respect to their weight,—i. e. the legs and feet,—the weight of the cinders of both bodies produced by the combustion was calculated not to exceed four pounds. The time which the combustion may have occupied could not have exceeded fourteen hours. These remains, lying upon a pavement covered by a greasy and stinking liquid, were surrounded by various pieces of furniture, &c. At the feet, the parts most distant from the fire was a table unburnt. The heads lay towards the hearth, in which there was no fire: a fender and andiron had fallen beneath the woman; and between her head and that of her husband was a brand, still burning. On the right was only a wooden shoe; on the left was a chair, one foot, four bars, and the straw cushion, which had been partially burned. There was also a bee-hive, reduced to a cinder. A few inches above the bodies was a besom made of rush, which was scarcely singed on one side, and some matches, the sulphurous end of which projected beyond a sabot which contained them.

[The circumstances attending these cases of spontaneous combustion agree generally with what has been hitherto observed in such cases. This account differs, however, from others, in two individuals, of different sex, being simultaneously affected; and it adds another fact to the rare occurrence of spontaneous combustion in man. It offers also an example of two individuals placed in such identical physiological conditions, that the combustion affected them both in the same degree and in the same parts.]

*Journal des Connaissances Médico-chirurgicales. Septembre*, 1836.

---

### *Employment of the Root of the Elder in Scrofula and Leucorrhœa.*
By M. Delens.

These observations were communicated to the Society of Medicine at Paris, and concern the administration of a remedy much used by the ancients.

M. Delens first used the remedy in the case of a lady of scrofulous constitution, afflicted with a tumour of the jaw. Various remedies were tried without benefit, and at last this medicine was given, not to act upon the tumour, but to produce an effect upon the constitution, which was debilitated: it, however, in a few days caused the absorption of the tumour.

A case of leucorrhœa is related, in which it was equally successful. It has likewise been given in several cases resembling those mentioned, with decided effect. It is given in the form of infusion; from two to four drachms in four cups of water, reduced to three, and taken during the day.

*La Lancette Française. Novembre*, 1836.

*On the Employment of Creosote and Tar-water in Pulmonary Affections.* By M. PETREQUIN, D.M.P.

THE exhibition of these remedies was confined to three sets of cases,—chronic bronchitis, incipient phthisis, and confirmed phthisis; and a certain number of each class were treated with creosote, and others with tar-water, and the comparative results carefully noted.

The usual effects produced were, a sense of heat in the œsophagus, stomach, and intestines, and vomiting. In two cases where diarrhœa existed, it appeared to exert a beneficial effect, by rendering the discharges fewer; and in one case, after each dose, and accompanying the sense of heat in the stomach, there were rapid alterations in the capillary circulation, with a sense of tingling in the limbs. In seven out of the fifteen cases, the cough and expectoration appeared to be lessened; but in two the irritation increased, and the cough became more frequent.

Upon the whole, M. P. has found the effects of creosote more beneficial in incipient than in confirmed phthisis; and in no case has he seen any thing approaching the radical cures announced by some. He concludes that it is only occasionally useful for the purpose of alleviation. He decidedly gives the preference to tar-water as a therapeutical agent. In one case, where the creosote produced great irritation, and rendered the cough worse and accompanied with vomiting, the tar-water had quite a different effect. After ten doses, the cough and dyspnœa became less, expectoration diminished, the pains in the chest were relieved, the nausea and vomiting vanished, and the patient went home well.

The creosote usually facilitated or diminished the expectoration; whereas, the tar-water always did so, without producing any of the unpleasant effects of the former.

The creosote sometimes alleviated the cough, at other times it appeared to exert no influence, and in two cases it rendered it more severe: on the contrary, the tar-water constantly alleviated it in a most striking manner.

The thoracic pains and sense of oppression were frequently diminished by the creosote; but the alleviation with the tar-water was constant.

As regards affections of the chest, tar-water certainly appears to be far superior to creosote; and, in its effects upon other parts of the system, the comparison is equally in its favour. The heat and irritation of the digestive tube, the disgust and vomiting, which were frequent attendants upon the action of creosote, did not occur during the exhibition of tar-water: indeed, it rather appeared to check vomiting.

Tar-water frequently increased the appetite, rendering the digestion more easy: no such effect was noted as regards creosote. Tar-water appeared to exercise no appreciable influence on the urinary secretion.

[One circumstance which strikes us as extraordinary in the above memoir is the smallness of the dose, which, in M. Petrequin's hands, produced nausea, and heat in the pharynx, œsophagus, and stomach. In the fifteen cases given, we find only one in which it reached five drops; whereas, in the remainder, these unpleasant symptoms were produced when the dose was only two drops. Dr. Elliotson states that many patients bear a gradual increase to ten or twenty drops, without unpleasant effects.] *Gazette Médicale de Paris, Novembre,* 1836.

---

*Chronic Fluor Albus, cured by Iodine.* By Dr. MÜLLER.

A YOUNG female had long suffered from leucorrhœa, which had diminished her strength, and had yielded to none of the means commonly employed in this disease; when the ointment of the hydriodate of potass was rubbed, morning and evening, into the internal surface of her thighs. After this inunction had been continued for four weeks, the disease had entirely ceased; and a careful and nutritious diet soon restored the strength.

*Wochenschrift für die gesammte Heilkunde,* No. 40. 1836.

*Clinical Researches concerning the Influence of certain Medicines upon the Functions of the Heart.* By M. Lombard, of Geneva.

[In a former Number we communicated some notices of M. Lombard's experiments with certain medicines on the action of the heart. He has undertaken some new researches regarding the therapeutical action of digitalis, assafœtida, camphor, and polygala senega, which we shall now briefly report.]

1. *Assafœtida.* M. L. states this to possess remarkable properties in combating the irregularity of the functions of the heart. Employed *externally*, in the form of plaster, it succeeds in alleviating palpitations which have resisted a great variety of medicines. He has almost constantly obtained some alleviation in a great number of cases. Irregular contractions of the ventricles, occurring in persons affected with disease of the heart, are modified; and it likewise succeeds in those cases which may be considered only nervous. The following is the formula used by him:

| | |
|---|---|
| Assafœtida, . . . | 2 ounces, |
| Gum. Ammoniac, . . | 1 scruple, |
| Turpentine, . . . | 6 drops, |
| Yellow wax, . . . | a sufficient quantity. |

Employed *internally*, he has found it likewise lessen and render regular the movements of the heart. In very small doses, it lessens the palpitations, and produces a remarkable calm; and he considers it a very valuable remedy in nearly all diseases of this organ.

2. *Camphor.* This medicine, given internally, in variable doses, from three to twelve grains in the day, acts in a special manner upon the heart. Among persons affected with hypertrophy, with dilatation of the cavities, the nervous influence is often insufficient to produce regular and complete contractions, and hence often tumultuous action. This state he has found can be modified by camphor, and he has seen the most tumultuous ventricular contractions become regular and perfectly isochronous after the administration of a few grains. He is not able to decide whether it acts as a stimulant or a sedative.

3. *Digitalis.* M. Lombard believes the want of uniformity in the sedative action of this medicine upon the functions of the heart, depends upon the four following circumstances: 1. The state of the stomach; 2, the mode of life of the patient; 3, the doses given; 4, the mode of administration. Sometimes, owing to an irritable state of the stomach, the exhibition of digitalis induces vomiting; and, if this continues after the cessation of the medicine, we must not have recourse to antiphlogistic measures, but to antispasmodics; such as the subnitrate of bismuth, oxide of zinc, and effervescing draughts. The mode of administering digitalis is one of the most important points in its therapeutical history. The infusion is the preparation which produces most promptly symptoms of saturation. In the form of powder, it rarely produces vomiting, except when the doses are large and frequently repeated. The best medicines for obviating or allaying these symptoms of saturation are calcined magnesia, subnitrate of bismuth, subcarbonate of iron, or oxide of zinc. M. L. considers the subcarbonate of iron as the best, and thinks he can attribute to its use the absence of baneful results among his patients who took digitalis daily for many months.

4. *Polygala Senega.* The therapeutical action of this medicine is little known. M. Lombard considers it one of the most precious which the materia medica possesses. Administered in the form of extract or infusion, he has found it lower the circulation, and especially regulate the ventricular contractions. The dose employed varied between twelve and twenty-four grains in the course of the day. The infusion, prepared with one drachm to four ounces of water, has been often administered in the same time.

*Bulletin générale de Thérapeutique, Nov.* 1836.

*Iodine in Mercurial Salivation.* By M. KLOSE.

SALIVATION had been produced in two children, during their convalescence, by mercury which had been administered on account of inflammation of the brain. To remedy the salivation, iodine was employed; and, after its first two doses, the peculiar smell of the mouth disappeared, the flow of saliva diminished, the pains became alleviated, and the aspect of the ulcers in the mouth was improved. The children were five and seven years of age. The iodine was discontinued before any of its peculiar symptoms were produced. M. Klose thinks iodine of value in such cases; and, as the remedies with which we are at present acquainted appear to possess but little influence over mercurial salivation, when it is once established, a new remedy which promises fairly is worthy of all acceptation.

*Medicinische Zeitung*, No. 34. 1836.

---

*On the Diuretic Operation of the Flowers of Statice Armeria.* By Dr. EBERS.

DR. EBERS speaks of the flowers of the Statice armeria, popularly termed "Pissblume" in Germany, as an active diuretic. From two drachms to an ounce of the flowers, freshly gathered and quickly dried, should be gently boiled, and the patient allowed to drink of the decoction, *ad libitum.* Some aromatic, as anise or cinnamon, is added to the decoction. The remedy appears to cause the excretion of urine in a direct manner. Dr. Ebers does not pretend to say the kind of dropsy to which it is appropriate, nor the mode of its operation.

*Wochenschrift für die gesammte Heilkunde,* No. 40. 1836.

---

*Intermittent Epistaxis, cured by Quinine.*

A STRONG man, æt. 27, suffered on alternate days from very violent bleeding at the nose, which continued from four to six hours, and could neither be put a stop to, nor alleviated by the common styptics, nor by any of the other means which are usually employed in similar cases. Regarding the periodicity of the occurrence of the bleeding, the treatment was changed, and a large dose of quinine, with diluted sulphuric acid, was administered. During the twenty-one days following, the bleeding recurred but twice, and was then readily stopped. The patient subsequently continued quite well. *Med. Zeitung*, No. 33. 1836.

---

*Cure of Hydrops Articuli, on the occurrence of Intermittent Fever.*

A MUSKETEER had been affected with dropsy of both knee-joints for a period of five months, and the disease had resisted a variety of treatment, which had been employed for its removal. At the end of this time he became the subject of a violent intermittent fever, a consequence of which was the cure of the effusion into the knee-joint; for, on the day following the appearance of the fever, there was no trace of fluid in either knee-joint.

*Medicinische Zeitung*, No. 37. 1836.

